# Head and neck cancer patient-derived tumouroid cultures: opportunities and challenges

**DOI:** 10.1038/s41416-023-02167-4

**Published:** 2023-02-10

**Authors:** B. W. M. Thilini J. Basnayake, Paul Leo, Sudha Rao, Sarju Vasani, Lizbeth Kenny, Nikolas K. Haass, Chamindie Punyadeera

**Affiliations:** 1 Saliva and Liquid Biopsy Translational Laboratory, The School of Environment and Science, Griffith Institute for Drug Discovery (GRIDD), Griffith University, Brisbane, QLD Australia; 2 School of Biomedical Sciences, Faculty of Health, Queensland University of Technology, Brisbane, QLD Australia; 3 Centre for Genomics and Personalised Health, Queensland University of Technology, Brisbane, QLD Australia; 4 Australian Translational Genomics Centre, Brisbane, QLD Australia; 5 Gene Regulation and Translational Medicine Laboratory, QIMR Berghofer Medical Research Institute, Brisbane, QLD Australia; 6 Department of Otolaryngology, Royal Brisbane Women’s Hospital, Brisbane, QLD Australia; 7 The School of Medicine, University of Queensland, Royal Brisbane and Women’s Hospital, Brisbane, QLD Australia; 8 Royal Brisbane and Women’s Hospital, Herston Queensland, Brisbane, QLD Australia; 9 Frazer Institute, The University of Queensland, Brisbane, QLD Australia; 10 Menzies Health Institute Queensland (MIHQ), Gold Coast, Griffith University, QLD Australia

**Keywords:** Head and neck cancer, Drug development, Preclinical research, Cancer models, Phenotypic screening

## Abstract

Head and neck cancers (HNC) are the seventh most prevalent cancer type globally. Despite their common categorisation, HNCs are a heterogeneous group of malignancies arising in various anatomical sites within the head and neck region. These cancers exhibit different clinical and biological manifestations, and this heterogeneity also contributes to the high rates of treatment failure and mortality. To evaluate patients who will respond to a particular treatment, there is a need to develop in vitro model systems that replicate in vivo tumour status. Among the methods developed, patient-derived cancer organoids, also known as tumouroids, recapitulate in vivo tumour characteristics including tumour architecture. Tumouroids have been used for general disease modelling and genetic instability studies in pan-cancer research. However, a limited number of studies have thus far been conducted using tumouroid-based drug screening. Studies have concluded that tumouroids can play an essential role in bringing precision medicine for highly heterogenous cancer types such as HNC.

## Head and neck cancers

Head and neck cancer (HNC) is an overarching term for a number of cancer types, categorised according to their anatomical sites, lip, oral cancer (OC), oropharynx cancer (OPC), larynx cancer, hypopharynx cancer, nasopharynx cancer, and thyroid cancer [1]. The most common malignancy of the upper aerodigestive tract is head and neck squamous cell carcinoma (HNSCC), which represents more than 90% of HNC [2]. HNC originating from salivary glands, soft tissues or nerves in the head and neck area are less common than squamous cell carcinoma. In 2018, HNC was the 7th most common cancer worldwide [3] and is often aggressive with high metastatic and recurrence rates [4]. Globally, there were 1.1 million cases in 2016, with 512,770 deaths, comprising 5.7% of global cancer-related fatalities [5]. HNC statistics demonstrate a predominance of OC in low- and middle-income countries, in which 67% and 82% of HNC cases and deaths, are reported respectively [6]. In Australia, in 2020, 5168 new cases were diagnosed, accompanied by 1151 deaths due to the disease [7]. According to global statistics, HNC is predominantly seen in men, which is two to four folds more than in women, estimating new cases over 20 per 100,000 [8]. According to The Lancet data in 2021, the average age of diagnosis of OC is 60 years, however recent data indicate that the OPC rates are increasing in people under 45 years old [9]. In addition to morbidity and mortality, HNC has a heavy burden on patients and their families as well as on the healthcare system due to late diagnosis [6].

Many risk factors contribute to the development of HNC. Smoking, betel nut chewing, chewing tobacco, and alcohol consumption are the primary risk factors for OC [10]. One of the major risk factors for OPC is infection by high-risk strains of Human Papillomavirus (HPV) [11–13]. HPV is primarily involved in cancers of the oropharynx, with the tonsils and the base of the tongue representing the most common subsites [14–17]. Expression of p16INK4A (p16 positive/cyclin-dependent kinase inhibitor 2 A, tumour suppressor protein) is highly correlated with HPV infection in HNC [18]. In addition, there are other risk factors associated with the development of HNC and these include poor nutrition, especially low vitamins A and B, poor oral hygiene, high consumption of salted food, high inhalation of hardwood dust (in sinus cancer), weakened immune system, and high radiation exposure [19]. Moreover, there are ethnic (e.g., Chinese) factors that promote the development of a subsite of HNC, nasopharyngeal cancers are primarily caused by Epstein-Barr Virus (EBV) [2, 7, 20, 21].

## Current treatment regimes

Current treatment for patients with HNC depends on the site where the tumour originates from. Treatments often involve a combination of surgery, radiation therapy coupled with chemotherapy, target therapy and immunotherapy. Early detection of HNC enables early intervention leading to better outcomes [2, 22]. The negative impact of treatment is high and often associated with considerable morbidity. Many patients, especially in low- and middle-income countries, must bear a substantial financial burden (financial toxicity) to receive the relevant health interventions, causing immense pressure on the patient and their families [12, 23]. In such countries, the primary mode of current treatment especially for patients with OC has been surgery as chemotherapy and radiotherapy are expensive and less readily available [13]. However, even for surgery, low- and middle-income countries often have limited capacity, with a relative paucity of surgical staff and a significant lack of healthcare facilities, resulting in a failure to receive timely and appropriate surgical care [12–14]. A prospective approach to finding better treatment options is to develop biomarkers to help choose drugs that are likely to be effective in patients with HNC. A number of diagnostic and prognostic biomarkers are currently being evaluated in clinical trials based on the REMARK guidelines, but their clinical significance is questionable [24]. In this section, we will focus on biomarkers that are currently being used for managing patients with HNC.

Chemotherapy stands as an important treatment option in HNC. Cisplatin is the widely used chemotherapeutic agent in HNC patients, and it is used either as a systemic single-agent or in combination with radiation therapy as a sensitiser. Cisplatin may also be used for the palliative treatment of HNC patients [25]. Cisplatin promotes DNA damage resulting in apoptosis in cancer cells as well as in normal healthy cells, where it is detrimental. Consequently, cisplatin is associated with marked toxicity, particularly with bone marrow suppression, renal damage, and ototoxicity. Toxicity is frequently dose-limiting. HNC patients who have comorbidities, such as hypertension, hyperlipidaemia, chronic obstructive pulmonary disease, renal failure, or diabetes, are at higher risk of suffering from side effects [6]. Many studies have been conducted to date to understand the mechanisms leading to chemoresistance to Cisplatin in cancers, but it is not yet fully understood [26]. Based on a meta-analysis using ten studies (sample size = 1317), Atashi et al. reported 33% cisplatin-resistance [27]. Since cisplatin is the first-line systemic treatment for HNC, it then becomes important to overcome cisplatin-resistance to improve prognosis [28].

Other standard chemotherapy regimens for stage III or IV HNC patients include 5-fluorouracil (5-FU), and docetaxel/paclitaxel, which can be used in combination with cisplatin [29]. In a total of 358 HNC patients, a combined strategy of docetaxel, cisplatin, and 5-FU (TPF) treatment significantly improved progression-free survival (11.0 months in TPF and 8.2 months in cisplatin and 5-FU) and overall survival (OS) (18.8 months in TPF and 14.5 months in cisplatin, and 5-FU) [30]. In late-stage HNC patients (*n* = 80), a combination of paclitaxel, cisplatin, and 5-FU (PPF) treatments yielded a 88% overall response rate and a 44% OS rate [31].

Cetuximab is a monoclonal antibody that targets Epidermal Growth Factor Receptor (EGFR), which has been approved by the Food and Drug Administration (FDA) in February 2004 [20, 22]. However, Cetuximab and other EGFR-targeting therapies have low efficacy, particularly in OPC. This may be a result of mutational changes in human epidermal growth factor receptors (HER), their ligands and other downstream signalling pathways [22]. Temporary inhibition of endocytosis promotes tumour cell antigen presentation, which in turn can enhance the efficacy of EGFR-targeted therapies [32]. However, further preclinical and clinical studies are needed to support the therapeutic value of this approach. Immunotherapy, one of the more recent treatment options, has garnered significant scientific and clinical interest. FDA has approved immunotherapeutic drugs, such as immune checkpoint inhibitors (anti-PD-1) namely Nivolumab and Pembrolizumab [33–36].

Over the past years, the standard of care for patients with HNC has rapidly evolved. A Chinese phase III randomised trial GEM20110714, demonstrated superiority in progression-free survival of gemcitabine/cisplatin over fluorouracil/cisplatin as first-line treatment for recurrent or metastatic nasopharyngeal carcinoma. After a median follow-up of 70 months, death occurred in 81.8% of patients in the gemcitabine/cisplatin group vs. 91.7% in the fluorouracil/cisplatin group, with a statistically significant hazard ratio (HR) of 0.72. The median overall survival was 22.1 months vs. 18.6 months, with 3- and 5-year overall survival rates of 31% vs. 20.4% (*P* = 0.021) and 19.2% vs. 7.8% (*P* = 0.001) [37]. Furthermore, a study linked to the Surveillance, Epidemiology, and End Results (SEER)-Medicare database, assessed a total cohort of 1395 patients for treatment responses, with 786 (56%) receiving cisplatin and 609 (44%) receiving cetuximab. The median follow-up period for those who survived was 3.5 years. HNC-specific mortality was significantly higher in the cetuximab cohort than in the cisplatin cohort (39% vs. 25% at 3 years: *P* = 0.0001). The adjusted hazard ratio of HNC-specific mortality for cetuximab was 1.65 (95% confidence interval, 1.30–2.09; *P* = 0.0001) relative to cisplatin in the matched cohorts (*n* = 414) [26].

## Treatments in clinical trials

In addition to established treatments, several drugs have recently been developed and are undergoing trials for HNC. These new treatments are currently in Phase 1 and Phase 2 clinical trials with most focusing on the development of targeted therapeutic agents, which can be used in combination with conventional therapies. Some of these targeted therapies that are in clinical trials include erlotinib, ABT-510 and bevacizumab, which are novel therapies for HNC.

Based on the clinical trial website (https://clinicaltrials.gov) [38], there are 2670 clinical studies recorded (to test current treatment combinations as well as novel treatments), while only 1085 were completed globally. Among the completed studies, 248 have shown favourable outcomes including improved overall survival (e.g.: Pemetrexed plus Gemcitabine), lower rate of recurrence (e.g., synergistic effect of Cetuximab, Hydroxyurea, Fluorouracil and radiotherapy), the low incidence rate of non-haematologic and haematologic toxicity side effects (e.g., synergistic effect of Kanglaite and chemotherapy) etc [38].

## Why are current treatments failing and why do HNC patients develop recurrences?

### HNCs are derived from diverse anatomical locations

Despite best efforts, the survival rates for HNC remain low, especially for p16-negative tumours. The relapse rate is high and there are many reasons that current treatments are frequently ineffective [1]. HNC is a highly curable disease when diagnosed at an early-stage, but early lesions are usually asymptomatic and often hidden due to their anatomical location [39]. The late diagnosis of the disease [40] leads to both poor prognosis with an average 5-year survival of < 50% and high healthcare expenditure [2]. Despite a combination of localised and systemic treatments, 40% of late-stage HNC patients do not respond or recur after first-line therapy. Within two years, 50% to 60% of these patients would have a loco-regional recurrence. Furthermore, 20% to 30% of those patients will develop distant metastases [41, 42].

### Relatively high tumour-genetic heterogeneity

HNC tumours are a heterogeneous group of tumours originating from the head and neck region and there are distinct subtypes [43]. Previous studies have bundled HNC into a single entity, also for therapy. This has led to treatment failures and loss of lives. We now understand that due to tumour heterogeneity, each subtype of HNC should be treated differently. As an example, oral cancer patients predominantly undergo surgery followed by chemoradiation whereas the early-stage of nasopharyngeal cancer is treated with radiotherapy as the primary and only curative treatment [9]. This is because each HNC subtype is anatomically, pathologically, and molecularly different, requiring more tumour agnostic-treatments.

Unlike lung cancer and breast cancers, HNCs do not have common tumour mutations “hot spots”. Most of the known genetic alterations are loss of function of tumour suppressor genes, such as TP53 (approximately 70% of all cases) [44] and p16INK4a (65% of all cases) [45] or activation of oncogenes such as EGFR (90% overexpressed in all HNC cases) [46] and PIK3CA (21% mutated in HNC cases) [22].

### Chemoresistance

Chemotherapy resistance has a significant impact on therapeutic efficacy and leads to poor prognoses in HNC patients [42]. Treating with cisplatin, 5-FU, and paclitaxel/docetaxel treatments cause four primary resistance mechanisms: DNA/RNA damage repair (cancer cell resist chemotherapy damage), drug efflux (reducing intracellular chemotherapy levels), apoptosis inhibition (cancer cell inhibits apoptosis protein), and EGFR/FAK/NF-κB activation (such signalling pathways promotes drug efflux, promotes cell proliferation, inhibits apoptosis) [29]. Different cellular biomarkers were identified related to the mentioned treatments. Examples of such markers are ERCC1 (causes DNA repair using cisplatin) [47], MDR1 (causes Drug efflux using cisplatin [48], paclitaxel [49] and docetaxel [50]), Livin (causes Apoptosis inhibition in using cisplatin and 5-FU) [51] and BST2 (causes EGFR/FAK/ NF-κB activation in using cisplatin) [52].

To overcome above mentioned challenges in current treatments, there is an unmet clinical need to develop preclinical model systems to accurately predict responses to the treatment of individual patients and to find biomarkers with high sensitivity and specificity taking into consideration tumour microenvironment (TME) interactions. Preclinical model systems would facilitate personalised treatment modalities for HNC patients [53], through the identification of HNC patients who are likely to respond to particular treatments before administration and saving patients from unwarranted toxicities.

## The need for in vitro model systems in cancer drug screening

In vitro model systems are important tools in cancer research to identify carcinogens, their involvement in molecular pathways during tumour growth and metastasis, and drug testing and development [54, 55]. In his third hallmarks of cancer paper, Hanahan proposed fourteen biological properties leading to the development of cancer, namely preserving proliferative signals, resisting growth suppressors, opposing apoptosis, enabling immortal replication, initiating angiogenesis, activating growth and metastasis, immunosuppressive nature of the tumour, inflammation in the tumour, changing cellular metabolism, genomic instability and mutation, epigenetic reprogramming, initiating plasticity and maintaining plasticity, cellular senescence and microbiome polymorphism [56]. Therefore, it is important to identify in vitro cell culture models that can accurately capture all if not most of these tumour activities. In vitro models for solid tumours range from 2D cancer cell lines to tumouroids [57]. The choice of the in vitro model systems depends on the research objectives [58]. For instance, pre-trial drug screening can be performed in 2D cell culture, whereas disease modelling (tumour growth/proliferation, migration, and invasion) and patient-derived cancer cell drug screening should be performed in tumouroids [59].

For every in vitro tumour model, the main component is the respective cancer cells themselves [60]. Cancer cell lines are easy to grow, and their molecular profile can be found in publicly available databases, e.g., the Cancer Cell Line Encyclopaedia (CCLE) [61, 62]. Cell types can vary from patient-derived cells, established cell lines, stem cells, immune cells, etc. For an appropriate cancer cell culture, factors such as biophysical properties, e.g., oxygen pressure, temperature, pH, condition of the extracellular matrix (ECM), and biochemical reagents need to be taken into consideration when developing in vitro culture assay models [54].

## 2D vs. 3D culture and spheroid vs. tumouroid culture

**Fig. 1:**
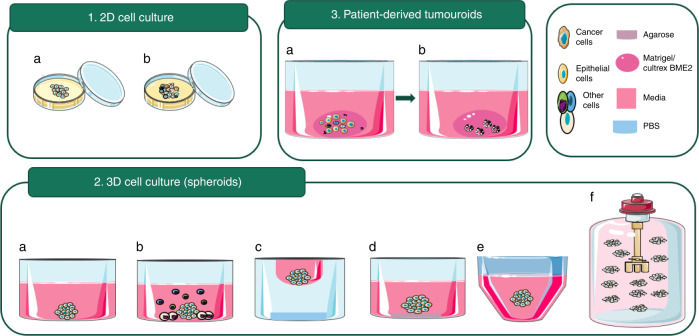
Different cell culture methods used in drug testing. **1a** Monolayer monoculture, **1b** monolayer mixed culture (e.g.: fibroblasts, mesenchymal stem cells), **2a** monoculture spheroids adherent to the flask, **2b** mixed culture spheroids adherent to the flask, **2c** spheroids in hanging drop method, **2d** spheroids suspended in a medium using 3D matrix e.g.: agarose, **2e** spheroids suspended in a medium using low attachment U bottom plates, **2f** spheroids in stirred systems, such as bioreactors, **3a** and **3b** Patient-derived tumouroids: Single cells derived from patient tumour samples and suspended in 3D matrixes such as Matrigel or Cultrex BME2.

In vitro cancer cell model systems initially evolved as 2D cultures. More recently, 3D culture systems have emerged (Fig. 1) [55]. In simplistic terms, 2D cell culture is grown either in suspension or adhesion to cell culture flasks [63]. As immortalised HNSCC cell lines are easily maintained and propagated, they have been widely employed to discover new molecular targets and novel small-molecular and biological treatments [64]. Such 2D cell culture systems are predominantly used by pharmaceutical and biotechnological companies as a preclinical method, as they are reproducible, cost-effective, amendable and can be used in high-throughput screening (HTS) [59]. However, the disadvantage of 2D cell monocultures is that they are unable to capture tumour architecture and the TME, which play a major role in how cells respond to drugs, and hence can be used in anti-cancer drug development [65]. For these reasons, stromal cells, such as fibroblasts and mesenchymal stem cells (MSC), have been incorporated into 2D cultures to account for complex cell-cell interactions in the presence of anti-cancer drug treatments [66, 67]. Cancer-derived fibroblasts can promote or inhibit cisplatin drug resistance in HNC cells [67]. Also, salivary gland cancer cells co-cultured with MSC showed more resistance to drugs such as paclitaxel and 5-aza-2’deoxycytidine [66]. However, even sophisticated 2D culture systems do not model the key fact that solid tumours develop in three dimensions (3D). Hence, 3D spheroid cell culture models were developed to test in vitro drug responses [43]. There were significant changes in drug sensitivity (IC_50_) between 2D and 3D culture from HNSCC cell lines when treated with cisplatin [68, 69], cetuximab [68, 69] and the mTOR inhibitor AZD8055 [64, 69]. Several strategies for culturing cells in 3D have been developed for drug screening studies in HNC. Some examples of 3D spheroid culture are adherent spheroids [70], hanging drop culture [71], non-adherent coating such as agarose [72], collagen or ultra-low attachment plates with round bottoms [68, 70] or growing 3D cultures in stirred systems, such as bioreactors [68]. 3D spheroid model systems are important for biopharmaceutical drug discovery because they are repeatable, robust, easy to utilise, may recapitulate the physiological microenvironment and are ideal for high-throughput screening [73]. However, a spheroid monoculture system lacks some of the tumour-stroma components and other cell types. Spheroids composed of tumour cells with stem cells [74, 75] or with cancer-associated fibroblasts [69] or fibroblast/epithelial cell (keratinocyte) monolayer have been used to solve this issue [76].

One of the reasons to mix different cell types is to enhance paracrine connections between tumour cells and stromal cells to mimic in vivo activities. These heterotypic cell-cell interactions have been shown to create more compact 3D spheroids and alter tumour cell intercellular communication and gene expression, resulting in altered tumour cell proliferation and migration [75, 76]. Such spheroid cell culture model systems are usually produced from established cell lines and as such are unable to recapitulate the true tumour, and cell-cell complexity, thus hindering the personalised approach [77].

**Fig. 2:**
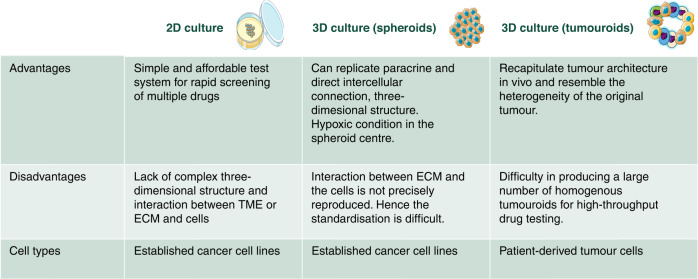
Comparison of 2D cell culture, spheroid, and tumouroid/organoid. Advantages, disadvantages, and cell types used in culture.

Patient-derived organoids/tumouroids are a possible solution to the issues that we have seen when using spheroid culture or 2D cell culture for drug testing. This is mainly because cancer organoids/tumouroids can more faithfully mimic the tumour in vivo status. Colorectal cancer tumouroids were the first to be established and are currently the most widely studied model systems. Subsequently, other cancer types, such as liver, pancreatic, gastric, brain, prostate, ovarian, lung, and oesophageal cancer, have been researched establishing that tumouroids maintain fidelity to the primary tumours’ histopathological, genomic, and functional features. In addition, tumouroids can be cryopreserved and hence can be used for biobanking. This feature of the tumouroids will be described in detail later in the review section (Fig. 2).

## Tumouroids

Preclinical cancer research is routinely performed using immortalised human cancer-derived cell lines [78], but increasingly, patient-derived xenografts (PDXs) are being used as they maintain the original tumour heterogeneity and tumour–stroma interactions [79, 80]. However, generating a PDX is a lengthy and costly process [81]. Tumouroids have been developed to address the limitations of using PDXs. The results of both assays were comparable; however, tumouroid drug screenings are more amenable to standardisation and can be performed routinely, in a shorter time frame, and with a limited amount of tissue derived from patients [80, 82]. Most human tumouroids are derived from treatment-naive primary tumours from patients who undergo the surgery before radiotherapy and any systemic treatments. Other research has used pluripotent stem cells (PSCs) or adult stem cells (ADSCs) [83], mutated cells to generate tumour models by manipulating genes by methods such as Clustered Regularly Interspaced Short Palindromic Repeats (CRISPR) [84], gene transfer [85] and RNA interference methods [86].

To understand the genetic heterogeneity of the tumours, in particular as part of disease modelling, the research focus has been on the genetic and epigenetic status of tumouroids/organoids. Genetic alterations that are present in tumours are captured using tumouroids, and as such, they have been used to determine a patient’s progression of cancer and response to treatment [87]. As an example, in colorectal cancer, driver mutation genes such as APC, KRAS, TP53, SMAD4, Wnt, and PIK3CA can be found in colorectal tumouroids [88].

Research into liver tumouroids has found mutations in CCND1 and CDKN2A genes, associated with the cell cycle, as well as genes associated with chromatin remodelling (ARID1A and ARID2) [89]. In bladder cancer, tumouroid research has found mutations in TP53 and FGR3 [90]. Tumouroids can maintain the heterogeneity of the original tumour, even after 16 passages [77]. This is an important aspect when using tumouroids. With this feature of recapitulating the original tumour, tumouroid research has shed light on identifying known mutations and new genetic variations during the progression of the tumours using whole-exome sequencing (WES). Even with several passages, tumouroids have similar mutations to their original tumour [91], highlighting that these can be used as reliable preclinical models. A strong clonal dynamic in tumouroids has recently been described, leading to pre-existing minor subclones [92]. The inherent genomic instability of cancer cells, as in all other models, is likely to result in completely novel genetic alterations during the continuous propagation of organoid models. This has been shown in different cancer types, namely kidney [93], colorectal [94], prostate [95], liver and pancreas [96]. As matched genomic data from multiple time points throughout tumouroids passaging become available, future studies will be required to characterise the extent of genomic evolution in cancer organoids. Therefore, tumouroids are useful model systems to find biomarkers for driver mutations that promote tumour growth and disease progression as tumouroid. This is due to the generation of a significantly large tumouroid collection (biobanking) that would increase the representation of rare genotypes as well as the statistical power to detect drug response molecular markers. Also, tumouroids have been used to identify the subclonal heterogeneity, which is the main cause of the resistance to modern anti-cancer treatments.

**Fig. 3:**
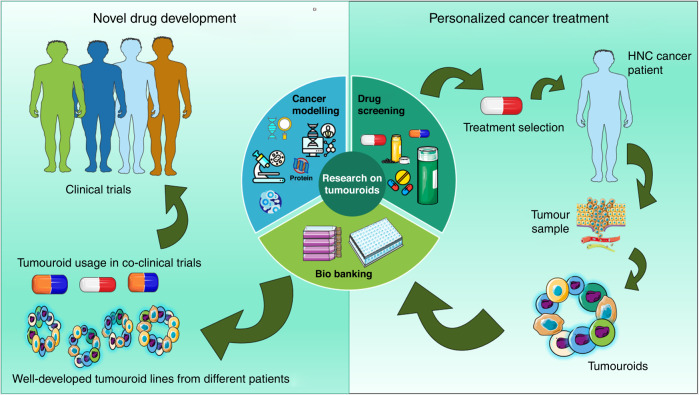
An overview of the applications of (toxicity evaluation of drugs, development of novel therapies through screening of drug targets and biobanking potentially leading to precision medicine approach). Tumouroids can be used for biobanking, a systematic and easy way to store patients’ clinical material for future research. Tumouroids can be characterised to identify biomarkers that play a role in cancer initiation and progression, cell origin, ability to drug resistance and connecting patient-specific genotype and phenotype (omics profiling—e.g., genomics, transcriptomics, proteomics, metabolomics).

Patient-derived tumouroids were initially used for disease modelling and for capturing genetic instability. Currently, tumouroids are used for screening for drug targets as well as testing drug efficiency (Fig. 3), allowing for precision medicine, which is tailoring disease prevention and treatment to individual differences in genes, environment, and lifestyle.

Earlier research relating to the organoid culture model (preclinical) was performed in 2009 by Hans Clevers and his team using Lgr5 + intestinal stem cells [97]. However, the first-time use of tumouroids for drug screening was performed by Van de Wetering et al. in 2015 [98]. Since then, drug screening using patient-derived tumouroids has had a significant advancement. Most of the research thus far is performed using colorectal tumouroids for drug screening. Van de Wetering et al. have achieved 90% efficiency in developing successful patient-derived colorectal tumouroids for drug screening. They have developed tumouroids in a 384-well format using luminescence-based cell viability read-out to report the drug susceptibility and such tumouroids were used in high-throughput drug screens. The findings also suggest the gene-drug association of tumouroids. For instance, tumouroids with TP53 mutations were resistant to nutlin3a (MDM2 inhibitor) and tumouroids with KRAS mutations were resistant to cetuximab (EGFR inhibitor). Similar research has been performed using breast cancer tumouroids [99]. Molecular and genetic similarities between the tumouroids and the original tumour were demonstrated, and those with BRCA mutations were sensitive to PARP inhibitors, which was consistent with clinical drug testing [100].

Pauli et al. have used several cancer types from different anatomical locations to establish tumouroids [101]. WES was performed to confirm genetic similarities between tumouroids and primary tumours (96%). In addition, there is a push to use known gene–drug associations (*n* = 160) in tumouroids using high-throughput drug screening with genomic analysis [101]. Similarly, some studies have predicted outcomes of drug treatments using genomic analyses of the cancer organoids [102].

Drug screening is one of the most important components of finding drug-related adverse reactions [103]. Studies have used organoids from healthy human organs such as kidneys, liver, and gut to verify drug resistance [104]. Additionally, studies conducted with gut organoids have been used to identify drug influx, efflux and metabolism, demonstrating the potential to determine the pharmacodynamics of drugs in the future [105].

Cancer heterogeneity has a great impact on treatment outcomes. As a result, precision medicine is becoming increasingly important, with the development of individualised cancer treatment plans based on increasingly specific prognostic markers and highly targeted therapies. Personalised tumouroids that are derived from individual patients can be used for genomic/transcriptomic testing [106]. Owing to the genomic complexity, there is a lack of understanding of pharmacogenomics in oncology [107]. Several studies have been conducted to find the effectiveness of personalised cancer medicine based on recapitulating genomic and histological features [108]. Well-designed patient-derived tumouroids can be the most useful tool for precision medicine, as tumouroids can be derived from a small tumour sample as well as different regions of the tumour, and are therefore able to screen for prognostic biomarkers, anti-cancer drugs, and optimising immunotherapy [109]. Cancer organoids can be used to define the mechanisms underlying immunity as the tumour may contain tumour-infiltrating lymphocytes and other immune cells, which make them recapitulate the key molecular and cellular features of primary or secondary tumours[110]. However, tumouroids’ lack of vasculatures limits their ability to be used as accurate models to study the effects of immunotherapies[111]. Complex cancer organoid models have been developed to overcome these limitations by co-culturing cancer organoids with immune cells [112], cancer-associated fibroblasts [113], and mesodermal progenitor cells [111, 114, 115]. Furthermore, co-culturing tumouroids with peripheral blood mononuclear cells or immune cells from lymph nodes can model cancer-immunity cycles such as the release of cancer cell antigens/cancer cell presentation, T cell priming/activation, T cell trafficking/infiltration into the tumour, T cell recognition/killing of cancer cells [111, 115, 116]. Additional supplements, such as anti-CD28, anti-CD3, and IL-2 antibodies, have been suggested for the long-term preservation of immune cells [111, 117]. Numerous clinical trials have been conducted to assess the various applications of tumoroids and their efficacy in precision cancer immunotherapy [111, 118]. The optimisation of drug screening platforms in terms of sensitivity and robustness is thus a critical aspect before based organoids-based models can be used in clinical practice [110].

One of the first papers published in the field of HNC-derived tumouroids was by Tanaka et al. [119]. They have introduced a method called cancer tissue-originated spheroids (CTOS), conducted based on a protocol developed by Kondo et al. [120]. The rest of the papers in this field were published by the Clevers and Driehuis groups [77, 121, 122] and the Kijima and Nakagawa research groups [123, 124]. Even though the Kijima and Nakagawa research group has focused mainly on oesophageal adenocarcinoma (EAC) and oesophageal squamous cell carcinoma (ESCC), they have claimed that this method can be adapted for use with HNC [123, 124]. Table 1 highlights the methods used for HNC tumouroid research.

**Table 1:** An overview of methods that have been used for head and neck cancer patient-derived tumouroids for drug screening.

Reference	Tumour tissues of origin	Tumouroids/ Organoids	Success Rate	Drug screening	Advantages		Limitations
Tanaka et al., 2018 [65]	Samples (*n* = 43) buccal mucosa, floor of mouth, gingiva, tongue, tonsil, pharynx, larynx	Cancer tissue-originated spheroids (CTOS)	30.2%	Cisplatin and docetaxel	CTOS method is reliable to generate tumouroids Tumouroids recapitulate tumour characteristics Drug sensitivity assay on tumouroids is similar to in vivo drug treatments.		The formation of tumouroids was high, however, growth rate and the ability to passage are much lower than the other cancer types. Continuous renewal and maintenance. The optimal condition is yet to discover.
Driehuis et al., 2019 [60]	Samples (*n* = 31) oral cavity (floor of mouth, tongue, and gingiva/alveolar process), pharynx, larynx, salivary gland, nasal cavity, and neck	Organoids from healthy mucosa tumouroids from cancer tissues	60%	Nutlin-3, Niraparib, everolimus, vemurafenib, alpelisib, cisplatin, carboplatin, cetuximab	Protocol for long expansion of tumouroids as well as organoids from healthy mucosa. Different drugs have been tested on tumouroids and received positive results. Tumouroids can be used along with ongoing clinical phase I and phase II.Genetic screening of the tumouroids can help to predict drug response.		Tumouroids can be improved by co-culture with immune cells in order to test immune therapies. More prognostic biomarkers are needed.
Driehuis et al., 2019 [67]	Samples (*n* = 11) tongue, larynx, oral cavity, parotid gland, gingiva	Organoids from healthy Mucosa Tumouroids from cancer tissues^a^	60%^a^	7D12, 7D12-9G8 and cetuximab	Characterising EGFR expression in HNSCC patient-derived tumouroids and treated with EGFR-targeting agents.		Biparatopic nanobody has a higher effect on HNC cells compared to monovalent nanobodies due to the small dimensions of tumouroids and lack of systemic distribution.
Kijima et al., 2019 [68]	Samples (*n* = 21) oesophageal and oropharyngeal squamous cell carcinomas^b^	Tumouroids from cancer tissues	71.4%	5-fluorouracil	CD44 expression plays a major role in tumouroid formation and the resistance for 5-fluorouracil. Tumouroid formation can be initiated with a small sample and small cell count Rapid growth (10–14 days) May predict patient therapy response Self-renewing cells at the passages Recapitulate the key features of the primary tumour. Laboratory and Pharmacological assays Translatable in personalised medicine.		Cell viability Fungal contamination Lack of influence of the tumour environment may influence IC_50_ Some patient samples may not support media/cell culture conditions Media/ cell culture conditions are not specific to cancer cells Tumouroid features may be different from the well-differentiated tumour Long-distance transportation and long-term cycro-preservation may alter tumour/ tumouroid features.
Driehuis et al., 2020 [66]	The paper is focusing on the guidelines based on previously published protocols^a^				Published a detailed protocol including Material, Procedure, Troubleshooting and Timing.		Not mentioned.
Karakasheva et al., 2020 [47]	Sample (*n* = 31) oesophageal adenocarcinoma and oesophageal squamous cell carcinoma	Tumouroids from cancer tissues^a^/^b^	60%–80%	Paclitaxel, 5-Fluorouracil, Cisplatin	Comprehensive protocol from generating tumouroids to the evaluation of the drugs on the tumouroids.		Signalling pathways are yet to be identified to passage successfully.

^a^Followed the same protocol as their initial research paper.

^b^Oesophageal adenocarcinoma (EAC) and Oesophageal squamous cell carcinoma (ESCC) samples were used however, the authors stated that the same protocol can be used for HNC.

## Comparison of tumouroid protocols

### Sample collection

When developing tumouroids, it is important that the method that is used should not influence the in vivo tumour status. Tanaka et al. do not mention how their samples were collected [119]. However, studies listed in Table 2 emphasise the importance of appropriate sample collection and tissue processing protocols. In Clevers’ and Driehuis’s methods, tumour samples were collected in Advanced Dulbecco’s Modified Eagle Medium/Ham’s F-12 (Advanced DMEM/F12) with L-alanyl-L-glutamine, which is a dipeptide substitute for L-glutamine (1× GlutaMAX), and included Penicillin–streptomycin, HEPES, and Primocin [62]. A recent study published by the same group recommended the addition of Rho kinase (ROCK) inhibitor (Y-27632), which helps tumour cells to proliferate in organoid cultures [92]. They also discourage sample transportation in sterilised ice-cold PBS, as it can result in cell death. They highlight the importance of maintaining the viability of the tumour sample (pink in colour) during the collection and transportation. Clevers’ and Driehuis’s method has been able to keep viable organoids for up to 72 h at 4 ˚C [92]. However, Kijima and Nakagawa recommend the transportation of samples in wet ice (4 ˚C). For the overnight transportation in Basal medium containing DMEM/F12 with 1× GlutaMAX included with 4-(2-hydroxyethyl)-1-piperazineethanesulfonic acid (HEPES), Antibiotic-Antimycotic, and Gentamicin have been used.

**Table 2:** A comparison of HNC patients derived tumouroids culture conditions.

	Clevers and Driehuis’s method	Kijima and Nakagawa’s method
Base media	Advanced DMEM/ F12 (includes GlutaMAX, HEPES, Penicillin–streptomycin)	Advanced DMEM/F12 (includes GlutaMAX, HEPES, Gentamicin, and Antibiotic-Antimycotic)
Primocin	100 μg/mL	
Gentamicin		10 µg/mL
Antibiotic-Antimycotic		x1
RN conditioned medium (that includes R-spondin and Noggin)		2%
B27 supplement	1	x1
N2 (x)		x1
R-spondin (% v/v for CM or ng/mL for rec)	4% v/v	50 ng/mL
Noggin (% v/v for CM or ng/mL for rec)	4% v/v	
N-acetyl-l-cysteine (NAC) (mM)	1.25	1 ng/mL
Nicotinamide (NIC) (mM)	10 mM	
Human Epidermal growth factor (EGF) (ng/mL)	50 ng/mL	
3-(6-Methyl-2-pyridinyl)-N-phenyl-4-(4-quinolinyl)-1H-pyrazole-1-carbothioamide (A83-01) (μM)	0.5 μM	
Human Fibroblast Growth Factor 10 (FGF10) (ng/mL)	10 ng/mL	
Human Fibroblast Growth Factor 2 (FGF2) (ng/mL)	5 ng/mL	
Prostaglandin E2 (μM)	1 μM	
Forskolin (μM)	1 μM	
CHIR 99021 (C_22_H_18_Cl_2_N_8_) (μM)	3 μM	
Rho-associated kinase (ROCK) inhibitor Y-27632 (μM)	10 μM	10 μM

### Sample processing and culturing of tumouroids

In the CTOS method, Tanaka et al. washed the tumour tissue in HBSS (Invitrogen, Carlsbad, CA), followed by removing the necrotic tissue. Then the tumour tissue sample was minced mechanically into small pieces and again washed with HBSS. The minced specimens were digested with 0.28 units/mL of Liberase DH (Roche, Basel, Switzerland) and 10 μg/mL DNase I (Roche) in DMEM/Ham’s F12 medium (Wako Pure Chemical Industries, Osaka, Japan) under constant stirring for 2 h at 37 °C. The digested specimens were filtered progressively through a metal mesh with a 100 μm pore diameter (Sigma Aldrich) and a 40 μm mesh (BD Falcon, Franklin Lakes, NJ, USA). The collected fragments were grown in ultra-low attachment culture dishes (Corning, Corning, NY) for 24–72 h with StemPro hESC (Invitrogen) and 8 ng/mL bFGF (Invitrogen) to generate CTOS. CTOSs were transferred to Matrigel and cultured in a growth medium once the formation was completed. The authors mentioned existing CTOS cell lines could be cultured for more than five passages and STR profiling confirmed genetically unique cell lines. This statement leads to the question of whether CTOS has more spheroid qualities than tumouroid qualities as the COTS consists of genetically uniformed cell lines. The methodology is relatively simple; however, the success rate was 30.2% and lower compared to other methods.

Sample processing for both protocols stated in Table 2 starts with mechanical fragmentation of the tumour samples. For enzymatic digestion, Clevers used 12.5% Trypsin [77], whereas Kijima and Nakagawa used a mixture of Collagenase IV, Y-27632, and HBSS-DF (HBSS-DFCY) to digest the tumour sample, and later 0.25% trypsin with DNase I was added for further digestion [123]. Both methods use a 100 µm strainer to filter the cells from the mixture [77, 123]. Clevers suspended cells in Cultrex growth factor reduced BME type 2 [77], whereas Kijima and Nakaga used Matrigel [123]. The organoid culture components are different in each method (Table 2). After establishing the organoid culture, both teams passage the organoids within 7–14 days and change media within 2-3 days. Considering the efficiency of these methods both claim to have 60–80% success rates [77, 121, 123].

### Drug screening and relevant Biomarkers

Tanaka et al. have evaluated organoids’ and their corresponding cell lines’ response to cisplatin and docetaxel to determine if organoids are suitable models for drug studies. Significantly, they have created an organoid (MDA-HN-2C) from a relapsed patient who underwent treatments with radiotherapy, cisplatin, docetaxel, and cetuximab. MDA-HN2016-2, a cell line established from the MDA-HN-2C organoid, has the highest IC_50_ compared to other cell lines. Also, the authors mentioned that MDA-HN-2C demonstrated significant resistance to docetaxel compared to MDA-HN2016-2. They performed drug testing using another three organoid lines (MDA-HN-1C, -18C and -21C) demonstrating different sensitivities towards cisplatin. Overall, cisplatin IC_50_ for MDA-HN-1C, MDA-HN2016-2, -18 and -21 were 0.76 µmol/L, 0.80 µmol/L, 1.12 µmol/L and 0.42 µmol/L, and docetaxel IC_50_ were 1.57 nmol/L, 0.59 nmol/L, 0.49 nmol/L and 0.30 nmol/L. They have not assessed the genomic biomarkers of these patients or organoids, however, they demonstrated the difference in cisplatin sensitivity of these patients due to expressing wild-type p53 (higher sensitivity to cisplatin) compared to p53 null and mutant p53 bearing cells (less sensitive to cisplatin) [119].

Clevers’ and Driehuis’s HNC-derived tumouroid culture methods have been used to test the efficacy of current chemotherapy, radiotherapy and targeted therapies. Initially, they tested commonly used drugs such as cisplatin, carboplatin, and cetuximab. Later they used radiotherapy (Grey field) on organoids or a combination of chemo- and radiotherapy to assess synergy in organoids. They also included targeted therapies such as alpelisib (PIK3CA inhibitor), vemurafenib (BRAF inhibitor), niraparib (PARP inhibitor), everolimus (mTOR inhibitor), and AZD4547 (FGFR inhibitor) in HNC-derived organoid culture models measuring ATP levels by Cell Titer-Glo (3-D Reagent, Promega) and the luminescence methods (Spark multimode microplate reader, Tecan) to determine the IC_50_ of drugs [77]. Researchers have also used the Growth rate inhibition (GR) metrics method, coupled to the area under the curve (AUC) and IC_50_ measurements. As an example, drugs such as carboplatin and alpelisib were used to demonstrate the drug sensitivity of HNC tumouroids. The drug screening methodology was similar to Clevers’ and Driehuis’s previously published research method [121].

Clevers’ and Driehuis’s HNC-derived organoid/tumouroid protocols were developed using healthy, normal oral mucosa and tumour tissue or biopsy samples respectively. Protocols include tumouroids’ characterisation using histology, gene expression, and mutational profiles [77, 121]. They have provided the first 3D model study for Herpes simplex virus (HSV) and found keratinocytes are essential for virion production in human papillomavirus (HPV16) studies. In their study, they demonstrate that 50–90% of the tumouroids overexpress EGFR. However, EGFR is not an effective prognostic biomarker for Cetuximab [122]. Similarly, the presence of PIK3CA gene mutations did not correlate with the success of alpelisib treatment. They tested the use of vemurafenib in two tumouroid lines with BRAF mutations. Only one cell line showed increased sensitivity towards the drug. Other targeted therapies (everolimus, niraparib, and AZD4547) have been tested on a panel of tumouroids without mutations in PARP, MTOR, and FGFR and produced various sensitivities towards the therapies. They suggested that this may be due to downstream genetic activation interfering with the action of target therapy [121]. Clevers et al. also attempted to establish tumouroids with cocultures of immune cells that provide 3D tissue architecture for drug treatments [77].

Kijima and Nakagawa used an HNC-derived tumouroid culture model system to determine the drug response of cisplatin and paclitaxel using the Cell Titer-Glo 3D method [123, 124]. Similar to Clevers’ and Driehuis’s method, Kijima and Nakagawa highlighted the importance of using tumouroids in a high-throughput setting to test drug sensitivity [124]. Kijima et al. have used CD44 as a drug target biomarker because CD44 is highly expressed on the tumour cell surface compared to normal mucosa. They have shown that Fluorouracil (5Fu) chemotherapy reagent has a higher resistance for CD44 expressed cells. They also emphasise the role of the tumour environment, which encompasses immune cells, endothelial cells, and cancer-associated fibroblasts. Furthermore, the same group has suggested that in future clinical applications, it would be beneficial to analyse precancerous stages as well as metastatic lesions [123].

## Limitations of tumouroids

Organoid/Tumouroid culture model systems have clear advantages over 2D and 3D cell culture systems, however, several limitations need to be addressed before clinical implementation. Firstly, it is important to establish tumouroids with minimal bacterial and fungal contamination, which may alter the response to drug treatments [125]. Secondly, there is a lack of standardised protocols for developing tumouroids. As an example, there should be a tumour-specific workflow for establishing tumouroids from the time of surgery until the tumouroid is transported and processed in the laboratory. More so, tumouroid culture depends on the condition of the tumour sample at the time of culturing. In particular, increased time between tumour tissue collection and culturing negatively impacts tumour tissue integrity and cell viability [126]. Methodology, especially media and supplements, differs between laboratories even for the same type of cancer [50].

It is known that tumouroids mimic the tumour microenvironment better than 2D cell culture, however, tumouroids lack vascular and neuronal networks. More so, the absence of increased interstitial pressure in tumouroid cultures may result in variations, which may influence drug screening [127, 128]. Also, the heterogeneity of the tumour tissue samples derived from cancer patients may contribute towards further variability and may affect the reproducibility of tumouroids [126, 129]. The balance between costs and time to generate tumouroids vs. their inherent advantages is another reason for the current paucity of drug screening using tumouroids derived from HNC patients.

## Future outlook

From discovery and development through to FDA post-market drug safety monitoring, the typical development of a successful anti-cancer drug takes more than a decade and costs on average US$ 1 billion [130]. Only 5% of potential drugs (e.g.: Bleomycin Sulfate, Cetuximab, Docetaxel, Hydroxyurea, Nivolumab, Pembrolizumab) [38] will progress through to a lead drug, that can be developed in laboratories with Good Laboratory Practice or Good Medical Practice before the manufacturing phase [130]. This is largely due to the reliance on 2D cell culture models and animal models that only partially recapitulate cancer patients’ genomic and pathophysiological profiles, which could hamper clinical effectiveness and toxicity. To date, there is a significant gap between in vitro and clinical research, hence a robust effective cell culture method is much needed. Tumouroid culture may serve as an effective in vitro model for drug testing as tumouroids recapitulate the 3D cell and tissue architecture of tumours and maintain the original tumour’s heterogeneity.

When using patient-derived tumouroids, they can be categorised according to anatomical location, genetic constitution, and clonal heterogeneity of the tumour for a better understanding of drug screening [131]. Compared to other tumour types, there are no common, functional ‘hot-spot’ mutations for HNC, and this has had a negative impact on drug development. Therefore, it is important to develop a culture model with individualised tumouroids, integrated with an effective workflow from tumour tissue collection to culture. The first step towards developing tumouroid culture is to establish a protocol, which sufficiently addresses key aspects including culture conditions, removal of contaminating cells and characterisation protocols.

It is also important to determine potential genomic/epigenomic biomarkers prior to drug testing in highly heterogeneous cancers, such as HNC. Therefore, patients’ tumours and tumouroids can be genetically analysed via DNA and RNA sequencing. In drug screening, these data can be used for preclinical trials as well as co-clinical trials where preclinical studies and clinical trials are simultaneously conducted. Genetic and transcriptomic data may translate into identifying a better biomarker for HNC drug treatment in future. When a patient enrols in a cancer clinical trial, a normal oral mucosal sample and tumour sample can be taken. From these samples, organoids, as well as treatment naïve tumouroids, can be established. Drugs can be administered to organoids and tumouroids to determine the patient’s response to treatment. Tumouroids can be used to identify the pharmacodynamics of the drug in conjunction with organoids, which can be used to identify dose-limiting toxicity. If the drugs show a high efficiency on the patient-derived organoids and tumouroids, patients could continue with the clinical trials. In a scenario where the drugs demonstrate low efficiency, the patient could be removed from the clinical trials. Also, when tumour tissue is available post-treatment (e.g., surgery after patients undergo chemotherapy), tumours can be harvested and grown into post-treatment tumouroids, which can be further used for drug sensitivity or resistance mechanism experiments. Tumouroids derived from post-treatments can be used to test alternative drugs, single-agent or combinations to understand the synergic effects of cancer therapies, which could be helpful to create alternative therapeutic regimens.

Tumouroids have the potential to be a powerful tool for tailored cancer therapy for patients. This method enables the creation of laboratory models directly from patient tumour tissue, eliminating the need for previous alteration or transformation (e.g., understanding the patient’s genomic profile). This leads to a highly personalised in vitro model that replicates the tumour tissue’s 3D architecture, morphology, physiopathology, and responsiveness to therapy in vivo, essentially replicating the patient in the preclinical environment and tumour heterogeneity and helping to select the best treatment for each patient.

Tumouroid culture model systems have been further developed using different methods, such as microcarriers [34], air-liquid interface (ALI) method [35, 36], Microfluidic device, Organoid-On-A-Chip models [28, 34], and organoids with bioreactors [34, 37]. These methods are currently in a development stage, with a significant focus on the extracellular environment including vascular, neuronal, and immune system input.

Despite these challenges, there is strong evidence that tumouroids can be used as a robust preclinical tool for drug screening, precision medicine, and developing anti-cancer drug treatments.

## Data Availability

Not applicable.
